# Reassortment and Mutations Associated with Emergence and Spread of Oseltamivir-Resistant Seasonal Influenza A/H1N1 Viruses in 2005–2009

**DOI:** 10.1371/journal.pone.0018177

**Published:** 2011-03-31

**Authors:** Ji-Rong Yang, Yu-Cheng Lin, Yuan-Pin Huang, Chun-Hui Su, Je Lo, Yu-Lin Ho, Ching-Yuan Yao, Li-Ching Hsu, Ho-Sheng Wu, Ming-Tsan Liu

**Affiliations:** 1 Research and Diagnostic Center, Centers for Disease Control, Taipei, Taiwan, Republic of China; 2 School of Medical Laboratory Science and Biotechnology, Taipei Medical University, Taipei, Taiwan, Republic of China; Louisiana State University Health Sciences Center, United States of America

## Abstract

A dramatic increase in the frequency of the H275Y mutation in the neuraminidase (NA), conferring resistance to oseltamivir, has been detected in human seasonal influenza A/H1N1 viruses since the influenza season of 2007–2008. The resistant viruses emerged in the ratio of 14.3% and quickly reached 100% in Taiwan from September to December 2008. To explore the mechanisms responsible for emergence and spread of the resistant viruses, we analyzed the complete genome sequences of 25 viruses collected during 2005–2009 in Taiwan, which were chosen from various clade viruses, 1, 2A, 2B-1, 2B-2, 2C-1 and 2C-2 by the classification of hemagglutinin (HA) sequences. Our data revealed that the dominant variant, clade 2B-1, in the 2007–2008 influenza emerged through an intra-subtype 4+4 reassortment between clade 1 and 2 viruses. The dominant variant acquired additional substitutions, including A206T in HA, H275Y and D354G in NA, L30R and H41P in PB1-F2, and V411I and P453S in basic polymerase 2 (PB2) proteins and subsequently caused the 2008–2009 influenza epidemic in Taiwan, accompanying the widespread oseltamivir-resistant viruses. We also characterized another 3+5 reassortant virus which became double resistant to oseltamivir and amantadine. Comparison of oseltamivir-resistant influenza A/H1N1 viruses belonging to various clades in our study highlighted that both reassortment and mutations were associated with emergence and spread of these viruses and the specific mutation, H275Y, conferring to antiviral resistance, was acquired in a hitch-hiking mechanism during the viral evolutionary processes.

## Introduction

Influenza viruses cause annual epidemics in many countries and occasional worldwide pandemics. Influenza vaccine and antiviral drugs are two useful measures for preventing influenza and reducing the impact of epidemics. Currently, there have been two classes of antiviral drugs available for preventing and treating influenza illness: M2 ion channel blockers, adamantanes (amantadine and rimantadine), and neuraminidase inhibitors (oseltamivir, zanamivir and peramivir). Since the 2005–2006 influenza season, most human influenza A/H3N2 viruses circulating worldwide were found to carry S31N in the M2 protein, conferring to adamantane-resistance [1], [2]. In the season of 2007–2008, oseltamivir-resistant seasonal influenza A/H1N1 viruses carrying H275Y (N1 numbering) in the NA protein were detected in Europe and spread globally in the absence of drug selective pressure [3], [4], [5]. At the end of the 2007–2008 season, the mean percentage of oseltamivir-resistant isolates was 25% in Europe, 12.3% in USA, 26% in Canada, 12% in Hong Kong and 3% in Japan [6], [7], [8], [9]. By the season of 2008–2009, most of the seasonal influenza A/H1N1 viruses have been resistant to oseltamivir [9]. Emergence and spread of antiviral-resistant viruses pose a challenge regarding the strategies for treating and preventing influenza.

There are three key factors affecting the evolutionary processes of antiviral resistant influenza viruses from their original appearance to worldwide spread. The first factor is the frequency of drug resistant mutants arising de novo during treatment, the second is the ability to overcome the obstacle of viral fitness caused by resistant mutations [10] and the last is to replace existing strains by reassortment and/or drift mutations. Based on the example of adamantane-resistant influenza A/H3N2 viruses, these viruses have been reported to occur de novo at a high frequency in about 27–33% of treated patients [11], [12] and these remained pathogenic and transmissible, compared to wild-type viruses [13]. The adamantane-resistant A/H3N2 viruses circulating worldwide in 2005–2006 were reported to be generated through reassortment and additional substitutions [14]. In the example of oseltamivir-resistant seasonal influenza A/H1N1 viruses, the frequency of drug resistant viruses appearing during clinical trials was from 0.3 to 18% [15], [16], [17]. Before 2006, oseltamivir-resistant seasonal A/H1N1 viruses were found to be compromised for replication and transmission *in vitro* and *in vivo*, compared to the corresponding wild-type [18], [19]. In the 2007–2008 influenza season, oseltamivir-resistant influenza A/H1N1 viruses likely adapted to the human population, became predominant and circulated globally. In particular, the viruses did not differ epidemiologically or clinically from susceptible isolates [3], [5], [20], [21], [22], [23]. Therefore, it is an important issue for understanding evolution of antiviral viruses that genetic change(s) may lead to optimization of viral fitness and enable a compromised virus to become predominant and spread globally.

In this study, we determined the frequency of the H275Y mutation in the NA to survey the oseltmivir-resistant seasonal influenza A/H1N1 viruses in Taiwan and found that a sudden and dramatic increase in the frequency of the H275Y mutation has been detected since September, 2008. Therefore, we combined the data of influenza epidemic surveillance as well as oseltamivir-resistance testing and complete genetic analysis of the circulating viruses during 2005–2009 to investigate the mechanisms responsible for emergence and spread of these resistant viruses in Taiwan.

## Results

### Increase of oseltamivir-resistant seasonal influenza A/H1N1 viruses in Taiwan from September 2008

**Figure 1:**
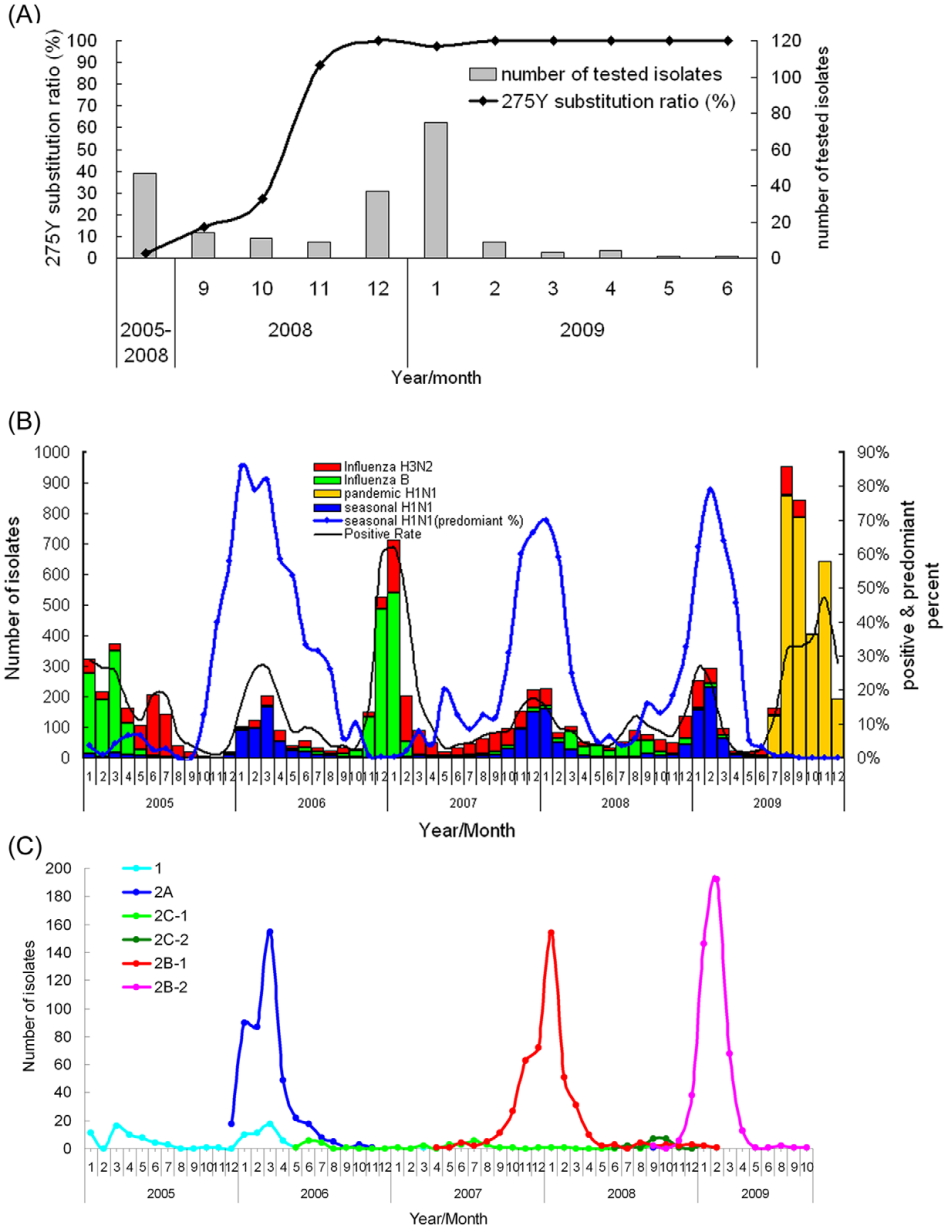
The frequency of the H275Y substitution in NA genes and monthly distribution of influenza isolates confirmed in Taiwan from 2005 to 2009. (A) The NA genes of seasonal influenza A/H1N1 viruses isolated in Taiwan from 2005 to 2009 were sequenced and analyzed for the mutation associated with oseltamivir resistance. The number of isolates tested and their H275Y ratio are illustrated by a black line and white bar, respectively. The H275Y positivity rate rose quickly from 4.2% (2/47) before August to 100% (37/37) in December in 2008. (B) The number of influenza isolates and their positivity rates. Seasonal influenza A/H1N1 viruses were the predominant circulating subtypes in the 2005–2006, 2007–2008 and 2008–2009 epidemics and the influenza B viruses were predominant in 2006–2007. The seasonal A/H3N2 viruses also co-circulated during these four influenza seasons. Since July 2009, the pandemic H1N1 viruses started to replace the seasonal influenza viruses and became dominant. (C) The clade-based epidemiological curves of the seasonal influenza A/H1N1 viruses. The 1585 isolates were classified into four distinct clades: 1, 2A, 2B (2B-1 and 2B-2) and 2C (2C-1 and 2C-2) according to the phylogenetic analyses and amino acid substitutions of HA sequences. The major viruses in 2005–2006, 2007–2008 and 2008–2009 seasons were clade 2A, 2B-1 and 2B-2, respectively. Minor species from other clades circulated during this period.

From January 2005 to August 2008, a total of 47 seasonal influenza A/H1N1 viruses in Taiwan were tested for the presence of H275Y substitution in the NA and only two (4.2%) isolates, A/Taiwan/0045/2006 and A/Taiwan/3293/2008, were shown to be positive for this substitution. However, the frequency of viruses harboring H275Y started to increase from September 2008 (2/14, 14.3%) and quickly reached 100% (37/37) in December (Fig. 1A). Because the seasonal influenza A/H1N1 viruses were the predominant subtype in the two successive influenza seasons of 2007–2008 and 2008–2009 in Taiwan (Fig. 1B), it was striking that the oseltamivir-resistant influenza A/H1N1 viruses emerged and spread quickly in the second (2008–2009) epidemic season.

### Variant evolution of seasonal influenza A/H1N1 viruses associated with the spread of oseltamivir resistance

From 2005 to 2009, three influenza epidemics in 2005–2006, 2007–2008 and 2008–2009 in Taiwan were caused predominantly by seasonal influenza A/H1N1 viruses (Fig. 1B). Among the total of 1660 viruses isolated during this period, we determined the partial HA sequences (HA1 domain) of the 1585 viruses. The epidemiological curves regarding various HA clade viruses were plotted in Fig. 1C. In Taiwan, the epidemic of 2005–2006 was dominated by clade 2A viruses, accompanied by clade 1 and 2C-1 viruses as minor variants. In addition, the clade 2C-1 viruses, which were first identified in May 2006, continued to circulate at low levels in the following years (Fig. 1C). The viruses of clade 2B-1 were first detected in April 2007 in Taiwan and peaked in January 2008, followed by transient replacement by clade 2C-2 viruses in September 2008. Another replacement occurred with viruses of clade 2B-2, which were first detected in September 2008 and quickly became the predominant strains from December 2008 (Fig. 1C).

To investigate the relationship between variant evolution and emergence of the oseltamivir-resistant viruses, we determined the prevalence of the H275Y substitution in various clade viruses in Taiwan. Of the HA clade 1 and 2A viruses, none was found to carry H275Y substitution (0/5 and 0/9, respectively; Table 1). Regarding the clade 2B viruses, 8 out of 34 (23.5%) clade 2B-1 viruses and all of the 130 clade 2B-2 viruses were positive for H275Y (Table 1). Among the 22 clade 2C viruses, there was only one belonging to clade 2C-2 detected with the H275Y (1/22, 4.5%, Table 1). Based on these comparisons, the high prevalence of oseltamivir-resistant seasonal influenza A/H1N1 viruses during 2008–2009 in Taiwan was mostly caused by viruses of clade 2B-2. This indicated that the emergence and predominance of this new variant was accompanied by the spread of oseltamivir resistance.

**Table 1:** H275Y substitution ratio of NA gene in different HA-clade influenza A/H1N1 viruses in Taiwan during 2005–2009.

residue 275 of NA gene	Clades (HA gene)					
	1	2A	2B-1	2B-2	2C-1	2C-2
H	5	9	26	0	6	15
Y	0	0	8	130	0	1
275Y ratio (%)	0	0	23.5	100	0	6.3

### Complete sequence analysis of the seasonal influenza A/H1N1 viruses

**Figure 2:**
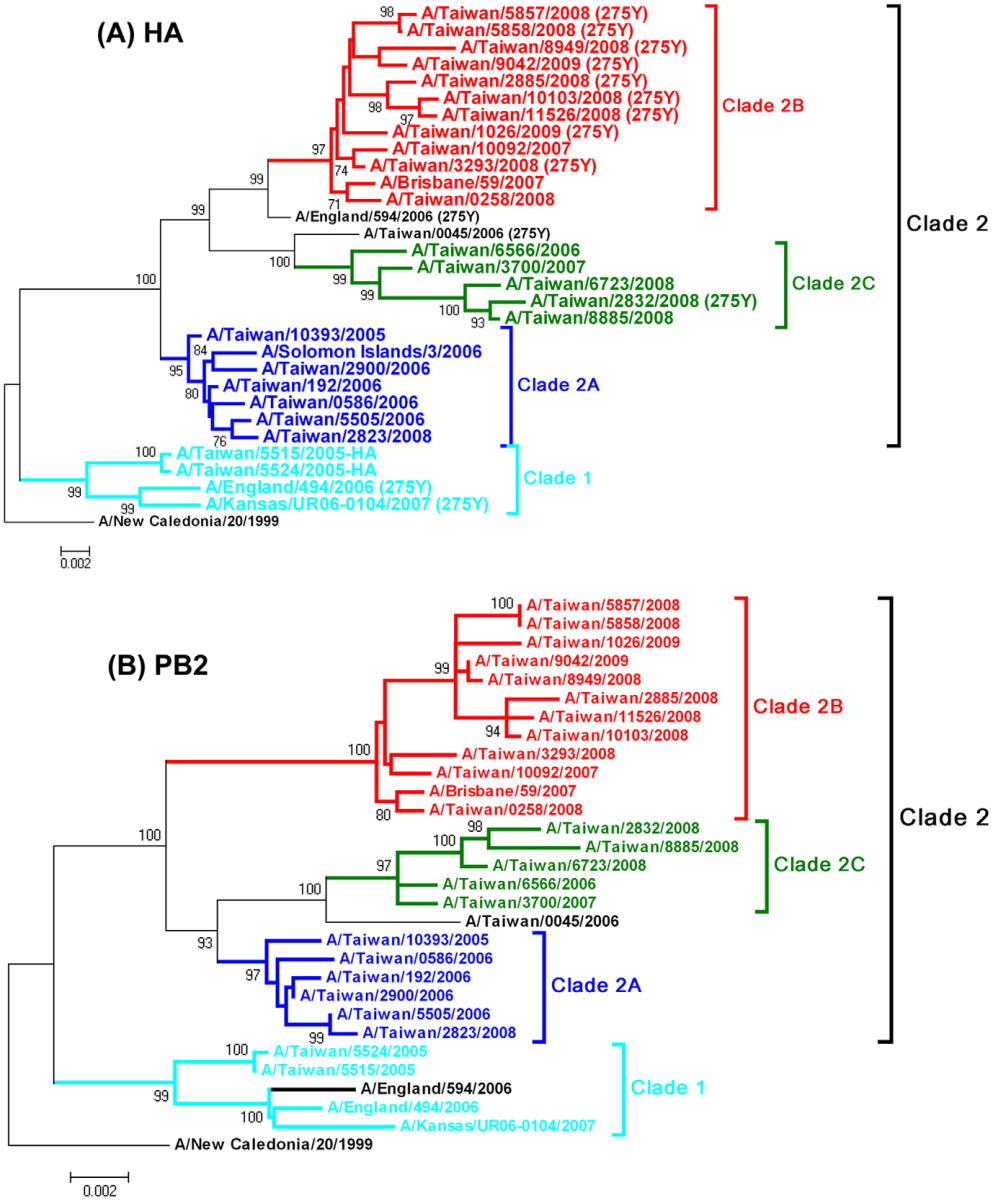
Phylogenetic relationships of the HA and PB2 segments of influenza A/H1N1 viruses in Taiwan. The phylogenetic analyses were constructed using the neighbor-joining method with 1000 bootstrap replications. Branch values of more than 75 are indicated. All of the phylogenies were rooted with the A/New Caledonia/20/1999, which was the vaccine strain recommended by WHO during the 2001–2007 influenza seasons. The genome sequences of A/Solomon Islands/3/2006, A/Brisbane/59/2007 and the early isolates carrying the H275Y substitution, A/England/494/2006, A/England/594/2006 and A/Kansas/UR06-0104/2007 obtained from the NCBI database also were included. Different clades were shown by different colors.

**Figure 3:**
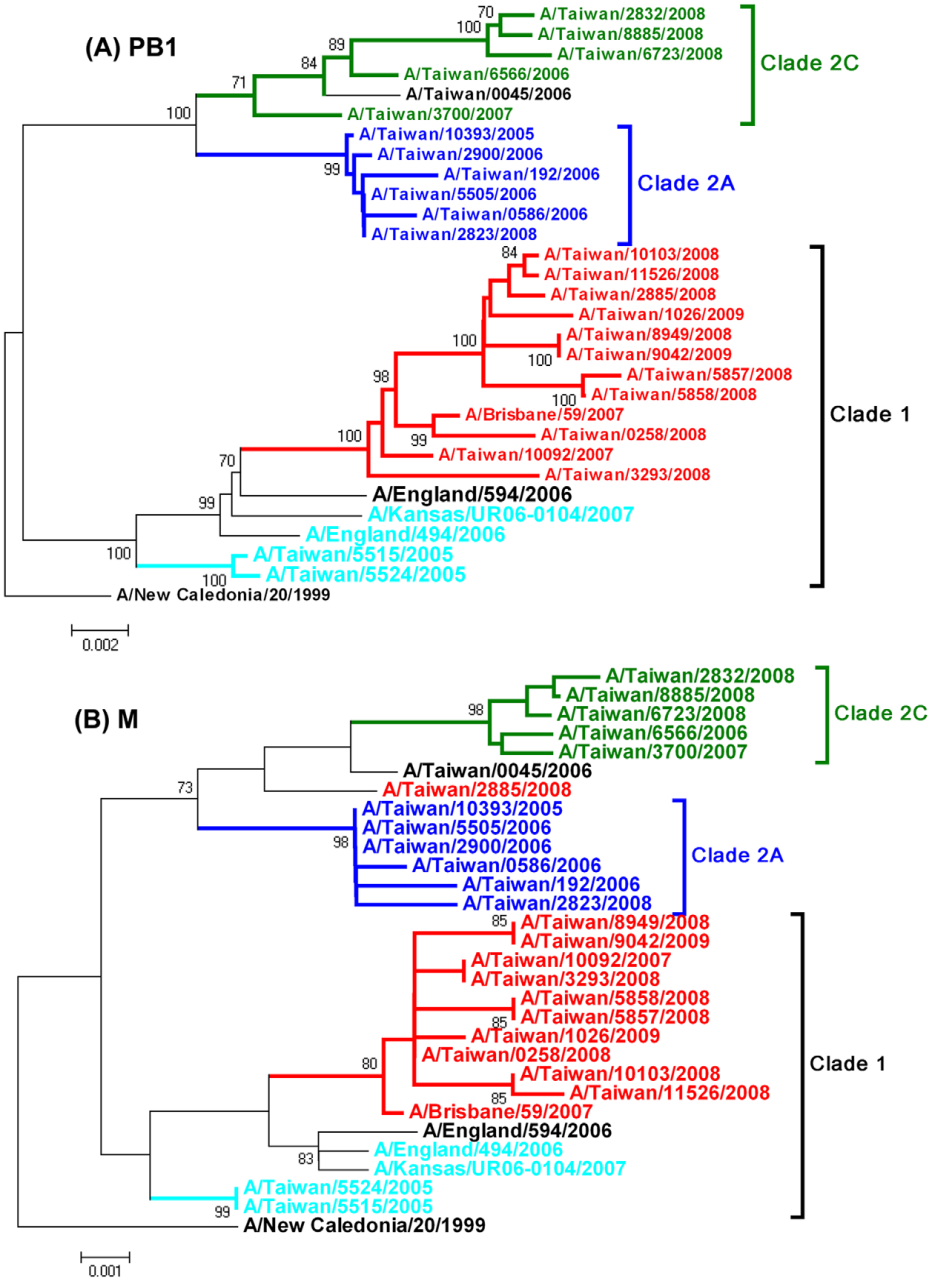
Phylogenetic relationships of the PB1 and M segments of influenza A/H1N1 viruses in Taiwan. The phylogenetic analyses were constructed using the neighbor-joining method with 1000 bootstrap replications. Branch values of more than 75 are indicated. All of the phylogenies were rooted with the A/New Caledonia/20/1999. Different clades were shown by different colors.

Twenty five representative viruses isolated during 2005–2009 in Taiwan, which were selected from different HA clade viruses (Table 2), plus three early oseltamivir-resistant influenza A/H1N1 viruses from other countries, A/England/494/2006, A/England/594/2006 and A/Kansas/UR06-0104/2007, and two vaccine strains A/New Caledonia/20/1999 and A/Brisbane/59/2007 were performed for complete sequence analysis (the last five were obtained from the NCBI database), and the phylogenetic topologies of each of the genome segments were determined. The phylogenetic topologies of HA, NA, PB2 and PA genes were similar. In the HA and NA trees, clade 2B viruses were more closely related to clade 2C than 2A (Fig. 2A and Fig. S1). In the PB2 and PA genes, clade 2A viruses were grouped with 2C rather than 2B (Fig. 2B and Fig. S1). In contrast, the phylogenetic analysis of PB1, NP, NS and M genes revealed that the clade-2B viruses classified by HA genes were shifted to clade 1(Fig. 3 and Fig. S2), except for A/Taiwan/2885/2008, whose M gene fell into clade 2 (Fig. 3B). The mismatch in these tree topologies strongly suggested that the viruses of clade 2B were generated through a 4 (PB1, NP, NS and M)+4 (HA, NA, PB2, and PA) reassortment between clade 1 and 2. The 8 segments of clades 2A and 2C were consistently located at clade 2, indicating that reassortment has not yet happened in these two clade viruses. In addition, the other two kinds of reassortment were observed. The virus A/England/594/2006 represented a 6+2 reassortment event between clade 1 and 2, that is, its PB2, PA, PB1, NP, NS and M segments were located in clade 1 and the HA and NA segments in clade 2 (Table 2). The virus A/Taiwan/2885/2008 represented a 3+5 reassortment event between clade 1 and 2. Its PB1, NP and NS segments were located in clade 1 and HA, NA, PB2, PA and M segments in clade 2 (Table 2).

**Table 2:** Characteristics of the 25 representative seasonal influenza A/H1N1 viruses analyzed in this study.

Virus name	Isolation date (y/m/d)	Clade classification in each phylogeny								Amino acid residue (of the gene)		Oseltamivir Carboxylate IC_50_ (nM)
		HA	NA	PB2	PA	PB1	NP	M	NS	275(NA)	31(M2)	
A/Taiwan/5515/2005	2005/1/13	1	1	1	1	1	1	1	1	H	S	1.02
A/Taiwan/5524/2005	2005/1/21	1	1	1	1	1	1	1	1	H	S	0.54
A/Taiwan/10393/2005	2005/12/28	2A	2A	2A	2A	2A	2A	2A	2A	H	S	0.37
A/Taiwan/2900/2006	2006/1/13	2A	2A	2A	2A	2A	2A	2A	2A	H	S	0.73
A/Taiwan/5505/2006	2006/1/4	2A	2A	2A	2A	2A	2A	2A	2A	H	S	0.49
A/Taiwan/0586/2006	2006/2/14	2A	2A	2A	2A	2A	2A	2A	2A	H	S	0.86
A/Taiwan/192/2006	2006/6/30	2A	2A	2A	2A	2A	2A	2A	2A	H	S	1.15
A/Taiwan/2823/2008	2008/9/19	2A	2A	2A	2A	2A	2A	2A	2A	H	S	0.57
A/Taiwan/10092/2007	2007/11/14	2B	2B	2B	2B	1	1	1	1	H	S	0.26
A/Taiwan/3293/2008	2008/5/2	2B	2B	2B	2B	1	1	1	1	Y	S	280.2
A/Taiwan/0258/2008	2008/10/16	2B	2B	2B	2B	1	1	1	1	H	S	0.36
A/Taiwan/10103/2008	2008/10/29	2B	2B	2B	2B	1	1	1	1	Y	S	153.4
A/Taiwan/2885/2008	2008/11/24	2B	2B	2B	2B	1	1	2	1	Y	N	177.3
A/Taiwan/11526/2008	2008/12/1	2B	2B	2B	2B	1	1	1	1	Y	S	159.2
A/Taiwan/1026/2009	2009/1/13	2B	2B	2B	2B	1	1	1	1	Y	S	156.5
A/Taiwan/9042/2008	2008/11/11	2B	2B	2B	2B	1	1	1	1	Y	S	139.9
A/Taiwan/5857/2008	2008/12/8	2B	2B	2B	2B	1	1	1	1	Y	S	347.9
A/Taiwan/5858/2008	2008/12/8	2B	2B	2B	2B	1	1	1	1	Y	S	821.7
A/Taiwan/8949/2008	2008/12/11	2B	2B	2B	2B	1	1	1	1	Y	S	471.3
A/Taiwan/0045/2006	2006/5/24	2	2	2	2	2	2	2	2	Y	N	358.4
A/Taiwan/6566/2006	2006/7/4	2C	2C	2C	2C	2C	2C	2C	2C	H	N	0.63
A/Taiwan/3700/2007	2007/3/19	2C	2C	2C	2	2C	2C	2C	2C	H	N	0.55
A/Taiwan/2832/2008	2008/10/6	2C	2C	2C	2C	2C	2C	2C	2C	Y	N	711.5
A/Taiwan/6723/2008	2008/10/13	2C	2C	2C	2C	2C	2C	2C	2C	H	N	0.35
A/Taiwan/8885/2008	2008/10/27	2C	2C	2C	2C	2C	2C	2C	2C	H	N	0.36
A/New Caledonia/20/1999*	1999/6/9	**-** #	**-**	**-**	**-**	**-**	**-**	**-**	**-**	H	S	ND
A/Brisbane/59/2007*	2007/7/1	2B	2B	2B	2B	1	1	1	1	H	S	ND
A/England/494/2006*	2006/4/11	1	1	1	1	1	1	1	1	Y	S	ND
A/England/594/2006*	2006/3/31	2	2	1	1	1	1	1	1	Y	S	ND
A/Kansas/UR06-0104/2007*	2007/1/30	1	1	1	1	1	1	1	1	Y	S	ND

*: Viruses as the reference strains.

# : A/New Caledonia/20/1999 was employed as an outgroup root for the trees.

ND: not determined.

### Amino acid substitutions associated with oseltamivir-resistant seasonal influenza A/H1N1 viruses

**Figure 4:**
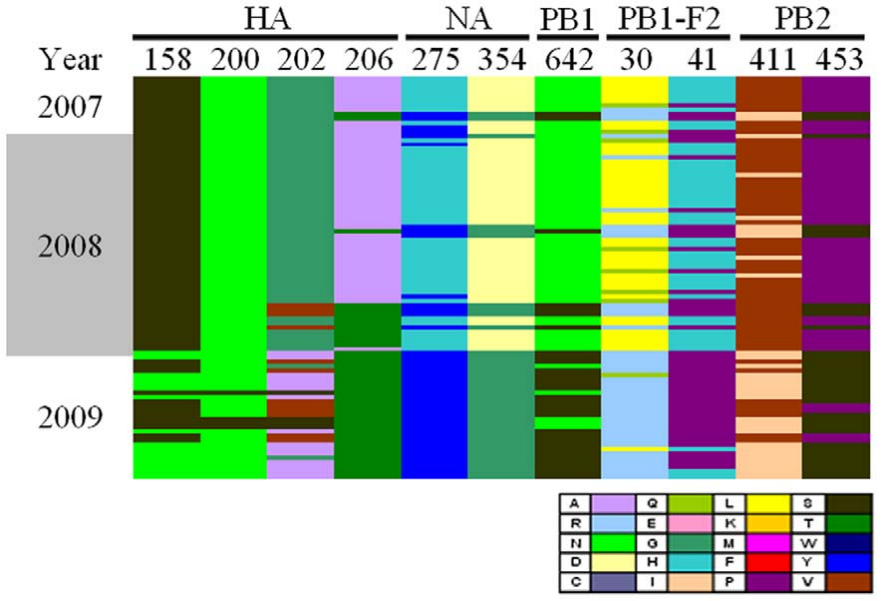
Amino acid changes in various proteins between clades 2B-1 and 2B-2 in the 92 global isolates. Amino acids substitutions at positions 158, 200, 202, 206 in HA, 275, 354 in NA, 642 in PB1, 30, 41 in PB1-F2, and 411, 453 in PB2 are represent by various colors. Each column represents the amino acid position indicated. Amino acids (single-letter abbreviations are used) are indicated by different colors, as shown in the key. Each row represents a single isolate and the 92 isolates analyzed are displayed in this figure in the order of collection time. The 92 isolate names are listed in Table S1.

To understand the genetic differences between the oseltamivir-resistant and sensitive viruses, amino acid substitutions of representative viruses in the same HA clade were compared. In clade 2B-1, there was only one virus with H275Y, A/Taiwan/3293/2008, identified before May 2008. It harbored only the H275Y substitution in the NA and no specific substitution was found by comparison with the wild-type clade 2B-1 viruses (Table 3). Of note, the other four clade 2B-1 viruses with H275Y, collected after October 2008 during the 2008–2009 epidemic, had five additional amino acid substitutions located in three different genome segments, including D354G in NA, L30R and H41P in PB1-F2, and V411I and P453S in PB2 (Table 3). Among the clade 2B-2 viruses in the 2008–2009 season, all of them harbored these five substitutions comparing to the wild-type clade 2B-1 viruses. We also detected additional substitutions at positions 158, 200 and 202 in HA (H1 numbering, referred to A/New Caledonia/20/1999 and the starting methionine counted as 1, used elsewhere in this study) and position 642 in PB1 genes (Table 3), which likely represented drift mutations and were associated less with the spread of clade 2B-2 viruses. To investigate whether these substitutions occurred in the global isolates, we analyzed extensively the 92 viruses belonged to clade 2B in HA genes isolated worldwide during 2006 to 2009 with available full genome sequences in the Influenza Virus Resource database (Fig. 4 and Table S1). Amino acid substitutions differences especially those between clade 2B-1 and 2B-2 were focused on and shown in Fig. 4. The substitutions, A206T in HA, D354G in NA, N642S in PB1, L30R and H41P in PB1-F2 and V411I and P453S in PB2 were also highly correlated with the H275Y in NA genes. Therefore, these widespread substitutions were observed in the viruses isolated from Taiwan as well as the global. For clade 2C viruses, the only one isolate with H275Y, A/Taiwan/2832/2008, was detected and isolated in October 2008. This had not acquired any additional substitution, compared to the wild-type clade 2C viruses (Table 3).

**Table 3:** Amino acid substitutions of the representative isolates in different proteins.

Virus name	Clade (HA)	Amino acid substitutions in various proteins														
		HA				NA					PB1	PB1-F2		PB2		M2
		158	200	202	206	194	222	234	275	354	642	30	41	411	453	31
A/New Caledonia/20/1999	-	S	N	G	A	G	R	V	H	G	N	L	H	V	P	S
A/Taiwan/5515/2005	1	S	N	G	A	G	R	V	H	G	N	L	H	V	H	S
A/Taiwan/5524/2005	1	S	N	G	A	G	R	V	H	G	N	L	H	V	H	S
A/Taiwan/10393/2005	2A	S	N	G	A	G	R	M	H	G	N	L	H	V	P	S
A/Taiwan/2900/2006	2A	S	N	G	A	G	R	M	H	G	N	L	H	V	P	S
A/Taiwan/5505/2006	2A	S	N	G	A	G	R	M	H	G	N	L	H	V	P	S
A/Taiwan/0586/2006	2A	S	N	G	A	G	R	M	H	G	N	L	H	V	P	S
A/Taiwan/192/2006	2A	S	N	G	A	G	R	M	H	G	N	L	H	V	P	S
A/Taiwan/2823/2008	2A	S	N	G	A	G	R	M	H	G	N	L	H	V	P	S
A/Brisbane/59/2007	2B-1	S	N	G	A	G	Q	M	H	D	N	L	H	V	P	S
A/Taiwan/10092/2007	2B-1	S	N	G	A	G	Q	M	H	D	N	L	H	V	P	S
A/Taiwan/3293/2008	2B-1	S	N	G	A	G	Q	M	Y	D	N	L	H	V	P	S
A/Taiwan/0258/2008	2B-1	S	N	G	A	G	Q	M	H	D	N	L	H	V	P	S
A/Taiwan/10103/2008	2B-1	S	N	G	A	G	Q	M	Y	G	N	R	P	I	S	S
A/Taiwan/2885/2008	2B-1	S	N	G	A	G	Q	M	Y	G	N	R	P	I	S	N
A/Taiwan/11526/2008	2B-1	S	N	G	A	G	Q	M	Y	G	N	R	P	I	S	S
A/Taiwan/1026/2009	2B-1	S	N	G	A	G	Q	M	Y	G	N	R	P	I	S	S
A/Taiwan/9042/2008	2B-2	N	N	A	T	G	Q	M	Y	G	S	R	P	I	S	S
A/Taiwan/5857/2008	2B-2	S	S	S	T	G	Q	M	Y	G	N	R	P	I	S	S
A/Taiwan/5858/2008	2B-2	S	S	S	T	G	Q	M	Y	G	N	R	P	I	S	S
A/Taiwan/8949/2008	2B-2	N	N	A	T	G	Q	M	Y	G	S	R	P	I	S	S
A/Taiwan/0045/2006	2	S	N	G	A	G	Q	M	Y	G	N	L	H	V	P	N
A/Taiwan/6566/2006	2C-1	S	N	G	T	G	Q	M	H	G	N	L	H	V	P	N
A/Taiwan/3700/2007	2C-1	S	N	G	T	G	Q	M	H	G	N	L	H	V	P	N
A/Taiwan/2832/2008	2C-2	S	N	G	T	G	Q	M	Y	G	N	L	H	V	P	N
A/Taiwan/6723/2008	2C-2	S	N	G	T	G	Q	M	H	G	N	L	H	V	P	N
A/Taiwan/8885/2008	2C-2	S	N	G	T	G	Q	M	H	G	N	L	H	V	P	N

We also analyzed the S31N substitutions in M2, conferring resistance to amantadine. Among the representative viruses, the eight viruses in HA clades 1 and 2A have no S31N substitutions. In clade 2B, only one isolate, A/Taiwan/2885/2008, was found to carry the S31N substitution. This double resistance to amantadine and oseltamivir resulted from the 3+5 reassortment of the virus (Table 2 and Fig. 3B). In clade 2C, all of the five isolates harbored the S31N substitution in M2 (Table 2).

### Neuraminidase activity and quasi-species analysis of the cultured seasonal influenza A/H1N1 viruses encoding 275H, 275Y in NA gene

The 50% inhibitory concentrations (IC_50_) for oseltamivir against the 25 representative viruses were determined (Table 2). Among them, the oseltamivir IC_50_ values of the 275Y encoding viruses were in the range 139.9–821.7 nM, while the values of the 275H ones were 0.26–1.15 nM. In addition, we examined the possibility if the IC50-tested sensitive or resistant viruses contained quasi-species of viral genotypes which were defined as components of 275H and 275Y populations in an oseltamivir-sensitive and resistant virus, by cloning and sequencing the PCR-amplified viral NA sequences. The sensitive virus, A/Taiwan/10092/2007, showed all 275H type (100%, 50/50) and also the resistant virus, A/Taiwan/3293/2008, showed all 275Y type (100%, 50/50), indicating quasi-species genotypes were not detected in these two viruses.

## Discussion

In this study, we provided a comprehensive analysis of the influenza A/H1N1 viruses collected in Taiwan during 2005–2009. The combined data of influenza epidemic surveillance, oseltamivir-resistance testing and complete genetic analysis of the circulating viruses during 2005–2009 revealed the evidence that the annual epidemiological replacement of various influenza A/H1N1 variants associated with emergence and spread of the oseltamivir-resistant influenza A/H1N1 viruses. In the early 2007–2008 influenza season, oseltamivir-resistant influenza A/H1N1 viruses were detected first in Europe (14%, 59/437), mainly in Norway (70%, 26/37). The frequency of oseltamivir-resistant viruses in Europe increased to 24.3% (727/2992) at the end of 2007–2008 influenza season. These viruses exhibited a NA H275Y substitution (N1 numbering), conferring resistance to oseltamivir and emerged without antiviral pressure [3], [7], [22], [24]. During April to September in 2008, the high frequency of H275Y substitution was detected in South Africa (225/225, 100%) and in Australia (93%, 71/76). In the same period, the high frequency of H275Y substitution has not yet been detected in Asia countries, including Hong Kong (17%, 97/583), Japan (14%, 1/7) and Taiwan (12%, 2/17) (in this study and [24]). In Japan, the ratio of resistant viruses increased from 0.4% (3/687) in the 2007–2008 season to 100% (745/745) in the 2008–2009 season [25]. In Americas, the frequencies of oseltamivir resistant influenza A/H1N1 viruses increased from 10.9% (111/1020, October 2007–May 2008) to 99.2% (649/654, October 2008–March 2009) in the United States of America (http://www.cdc.gov/flu/weekly/pastreports.htm). The global frequency of resistant viruses increased from 44% (April 2008–September 2008) to 96% (October 2008–March 2009) [9], [24]. Based on the tempo-spatial global distribution of oseltamivir-resistant influenza A/H1N1 viruses, spread trend of there resistant viruses seemed to start from Europe and migrated to the southern hemisphere, followed by North America and Asia. The emergence and fast global spread highlight the importance of continuation of antiviral resistant surveillance and timely global response.

Genome segment reassortment contributes to the increase of influenza diversity and is associated with severe epidemics and disaster pandemics. Influenza A viruses caused four human influenza pandemics in 1918 (H1N1 subtype), 1957 (H2N2 subtype), 1968 (H3N2 subtype) and 2009 (H1N1 subtype), and those viruses all emerged through complex inter-subtype reassortment events [26], [27]. Furthermore, intra-subtype reassortments as well as antigenic drift have been demonstrated to be an important process in the evolution and annual epidemics of human influenza A/H1N1 viruses and were responsible for several notable epidemics of unusual severity in the 1940s and 1950s [28]. In this study, we investigated the evolutionary histories and epidemiology of seasonal influenza A/H1N1 viruses in Taiwan. During 2005–2009, there were four influenza epidemics in Taiwan and three of the four were caused by the H1N1 subtype and predominated by different variants, including HA clades 2A, 2B-1 and 2B-2, respectively (Fig. 1C). The epidemic variant turnover from clade 2A to 2B-1 during 2005–2007 was due to the effect of the intra-subtype reassortment and drift mutations (Table 2 and 3). The following two successive influenza epidemics of 2007–2008 and 2008–2009 in Taiwan were predominated by variant clades 2B-1 and 2B-2, respectively, and they were antigenically relative to A/Brisbane/59/2007, the influenza A/H1N1 component of the 2008–2009 and 2009–2010 influenza vaccine for the Northern Hemisphere (data not shown and [29]). These two variants had similar genetic segments and differed by major mutations located in HA, NA, PB1, PB1-F2 and PB2 (Table 3). In addition, the non-reassortant clade 2C viruses, which could not become dominant and spread widely like clade 2B viruses, were also coexisting and circulating continually at low activity during 2005–2009 in Taiwan. These results strongly highlighted the importance of genome segment reassortment and drift mutations of surface and internal genes in the predominance and annual epidemic recurrence of influenza viruses.

Worldwide spread of antiviral-resistant influenza viruses has been observed for subtypes H3N2 and H1N1. Genomic reassortment and additional substitutions also have been considered as important in the evolutionary processes of emergence and spread of adamantane-resistant influenza A/H3N2 viruses in 2005–2006 and a hitch-hiking mechanism was used to explain the occurrence of the S31N substitution in M2 protein of this particular variant [14]. In this study, we observed that the worldwide spread of oseltamivir-resistant influenza A/H1N1 viruses during 2007–2009 were generated by reassortment and drift mutations, and the H275Y in the NA protein also seemed to spread in a hitch-hiking mechanism. We analyzed the phylogenetic trees of the four early oseltamivir-resistant viruses collected before January 2007 and the representative viruses isolated in Taiwan during 2005–2009. The resultant phylogenies revealed that each segment of A/Taiwan/0045/2006 was closely related to clade 2C and those of A/England/494/2006 and A/Kansas/UR06-0104/2007 were located in clade 1 (Table 2). Of note, the clade 2B-related A/England/594/2006, whose PB2 and PA genes still fell into clade 1, represented another reassortment event-a 6+2 pattern between clade 1 and 2 (Table 2). The data suggested that the resistant viruses prior to 2007 occurred sporadically in clade 1 and clade 2, and new variants (clade 2B) were generated through intra-subtype reassortment events between these two clades stepwise (6+2 to 4+4) during 2006–2007 and acquired additional substitutions in the evolutionary processes. During the epidemic turnover of dominant variants from clade 2B-1 to 2B-2 viruses within the two influenza seasons of 2007–2008 and 2008–2009 in Taiwan, several major substitutions were detected without genomic reassortment, including A206T in HA, H275Y and D354G in NA, N642S in PB1, L30R and H41P in PB1-F2 and V411I and P453S in PB2 (Table 3). Some of there substitutions should enhance the viral fitness and contribute to epidemic recurrence. In the previous studies, the residue 354 in NA protein was found to locate at the top of the neuraminidase tetramer, the residue 206 was the receptor binding domain of the HA1 protein and the residue 453 was the nuclear localization signal of the PB2 [20], [30], [31]. However, the roles of these substitutions on influenza epidemiology remain to be studied. It was worth mentioning that these substitutions were not observed in oseltamivir-resistant clade 2C viruses, except for A206T in HA and D354G in NA. These results revealed that the H275Y NA substitution occurred coincidentally with the advantageous substitutions and hitchhiked with a dominant variant during the evolution processes, resulting in the widespread resistant viruses.

Some substitutions R194G, V234M and R222Q of NA proteins were involved in buffering deficiencies on folding or stability of neuraminidase caused by the H275Y substitution and restore viral fitness [32]. In this study, all of 25 viruses in HA clades 1, 2A, 2B and 2C harbored the R194G substitution, and the viruses of the last three clades also carried the V234M substitution. For the R222Q substitution, the clade 2B and 2C viruses have acquired this substitution (Table 3). Overall, both clade 2B and 2C viruses have acquired sufficient changes to overcome the obstacles caused by H275Y. However, the clade 2C viruses were still not observed to spread widely like clade 2B viruses, indicating that these substitutions, buffering deficiencies induced by the H275Y substitution, were required but not enough for the widespread oseltamivir-resistant viruses. It was suggested that when a suitable genetic background was established through reassortment and mutations, the resistance-conferring mutation H275Y in the NA gene could hitch-hike with other compensating mutations elsewhere in the genome to make a dominant oseltamivir-resistant virus spread. In addition, since the double resistant viruses from various clade 2B and 2C viruses have been found in Taiwan, and also detected in Hong Kong [33], [34], the possibility for worldwide spread of multiple antiviral-resistant viruses should be concerned.

## Materials and Methods

### Collection of specimens, viral isolates, viral RNA extraction, and the medication of patients

This study was conducted through the national influenza surveillance network in Taiwan, which is coordinated by the Centers for Disease Control (CDC), Department of Health, Taiwan. This surveillance points cover approximately 75% of basic administrative units of Taiwan (cities, townships or districts) [35]. More than ten virology laboratories in Taiwan routinely collected clinical specimens throughout year and isolated viruses by using Madin Darby canine kidney (MDCK) cells and identified subtypes by immunofluorescence assay (IFA). Then, these isolates were sent to CDC, Taiwan for further analysis. Viral RNA was extracted from the supernatant of cultured viral isolates using QIAamp Viral RNA Mini Kits, according to the manufacturer's instructions (Qiagen, Santa Clara, CA). Automated extraction also was conducted using the MagNa Pure LC extraction system (Roche). For the medication of the patients with influenza-like illness, oseltamivir was rarely prescribed in Taiwan before May 2009 (the outbreak of 2009 pandemic H1N1). We believed that the analyzed viruses in this study were isolated from patients without oseltamivir-treatment, although we did not have their clinical record of drug therapy.

### Epidemic curve of various clade influenza A/H1N1 variants confirmed in Taiwan from 2005 to 2009

The partial HA sequences of the influenza viruses from 2005–2009 were determined by conventional RT-PCR and sequencer (ABI 3730). Based on phylogenetic analyses and amino acid substitutions of analyzed sequences, we classified these seasonal influenza A/H1N1 isolates into four distinct HA clades: 1, 2A, 2B and 2C, which were designated by a previous study [29]. The signature amino acids changes from clade 1 to 2 included T99K, K225R, F269Y and T283N of the HA gene (H1 numbering, referred to A/New Caledonia/20/1999 and the starting methionine counted as 1, used elsewhere in this study). For the clade 2, the key substitutions of clade 2B were D52N, K157E, R205K and E290K, and those for clade 2C were D53N, R205M and A206T. Clade 2B could be classified further into subclades 2B-1 and 2B-2, based on the additional substitution of A206T, specific for clade 2B-2, and also, clade 2C into 2C-1 and 2C-2 by additional substitutions I64K and E85G, carried by clade 2C-2 viruses.

### Antiviral resistance and complete sequence analysis of the seasonal influenza A/H1N1 viruses

Virus isolates identified as influenza A/H1N1viruses were tested for oseltamivir resistance by analysis of NA sequences through a conventional PCR. A 907 base-pair DNA fragment spanning nucleotide position 823–825 (amino acid position 275, N1 numbering) of NA gene was amplified and the nucleotide sequences also were determined. Isolates with a C to T substitution at nucleotide position 823 that could result in the substitution of histidine (encoded by CAC) for tyrosine (encoded by TAC) at amino acid position 275 in the NA protein were identified as oseltamivir resistant. To focus in more detail on the genetic diversity and phylogenetic histories of the oseltamivir-resistant seasonal influenza A/H1N1 viruses during 2005–2009 in Taiwan, 25 representative isolates belonging to various HA clades, 1, 2A, 2B and 2C, which were classified based on the HA sequences and designated according to a previous study [29], were selected for complete genome sequencing. The basic information for the 25 isolates is shown in Table 2. The three oseltamivir-resistant influenza A/H1N1 viruses, A/England/494/2006, A/England/594/2006 and A/Kansas/UR06-0104/2007, isolated before the 2006–2007 influenza season and with complete genomic sequences available from the NCBI influenza virus sequence database [36], also were included in the study as reference sequences of oseltamivir-resistant viruses in the early stage. Multiple sequence alignments, protein translation and phylogenetic analysis were performed on the basis of nucleotide sequences using the software MEGA4 [37] and BioEdit (http://www.mbio.ncsu.edu/BioEdit/bioedit.html). A phylogenetic tree was constructed by the neighbor-joining method and 1,000 bootstrap replications were performed to evaluate the robustness. The vaccine strains A/New Caledonia/20/1999 and A/Brisbane/59/2007 also were included in the eight phylogenies as reference sequences.

### NA inhibition assay of the cultured influenza A/H1N1 virus

The 50% inhibitory concentration (IC_50_) analysis of oseltamivir for influenza A/H1N1 viruses was determined using the NA-Star Influenza Neuraminidase Inhibitor resistance Detection Kit (Applied Biosystems) according to the manufacturer's recommendations. Briefly, 25 µl of half-log dilutions of neuraminidase inhibitor (NI) from 0.03 to 1000 nM were mixed with 25 µl of virus dilution with equal amount viruses of 16 hemagglutination (HA) unit in each well of a white 96-well microplate and incubated 10–20 min at 37°C. Two wells of the mixture destined to be negative controls contained only assay buffers, instead of NI, and culture medium, instead of viruses, also were included. Then, 10 µl of diluted substrate was added to each well and incubated for 10–30 min at room temperature, followed by the addition of 60 µl of accelerator, the chemiluminescent signal being measured immediately. The software GraphPad Prism version 4.00 was used to determine the IC_50_ values.

### Sequences information

The nucleotide sequences of influenza viruses in this study have been submitted to GenBank and their accession numbers are HQ291845-HQ292044.

## Supporting Information

Figure S1 **Phylogenetic relationships of the NA and PA segments of influenza A/H1N1 viruses in Taiwan.** The phylogenetic analyses were constructed using the neighbor-joining method with 1000 bootstrap replications. Branch values of more than 75 are indicated. All of the phylogenies were rooted with the A/New Caledonia/20/1999. Different clades were shown by different colors.
(TIF)

Figure S2 **Phylogenetic relationships of the NP and NS segments of influenza A/H1N1 viruses in Taiwan.** The phylogenetic analyses were constructed using the neighbor-joining method with 1000 bootstrap replications. Branch values of more than 75 are indicated. All of the phylogenies were rooted with the A/New Caledonia/20/1999. Different clades were shown by different colors.
(TIF)

Table S1 **Amino acid substitutions in various proteins, differing between clades 2B-1 and 2B-2 in the 92 global isolates.**
(DOC)
