# Supplementary figures and images for: Reassortment and Mutations Associated with Emergence and Spread of Oseltamivir-Resistant Seasonal Influenza A/H1N1 Viruses in 2005–2009
Source: PLoS One. 2011 Mar 31;6(3):e18177. doi: 10.1371/journal.pone.0018177 (PMC3069057; doi:10.1371/journal.pone.0018177)

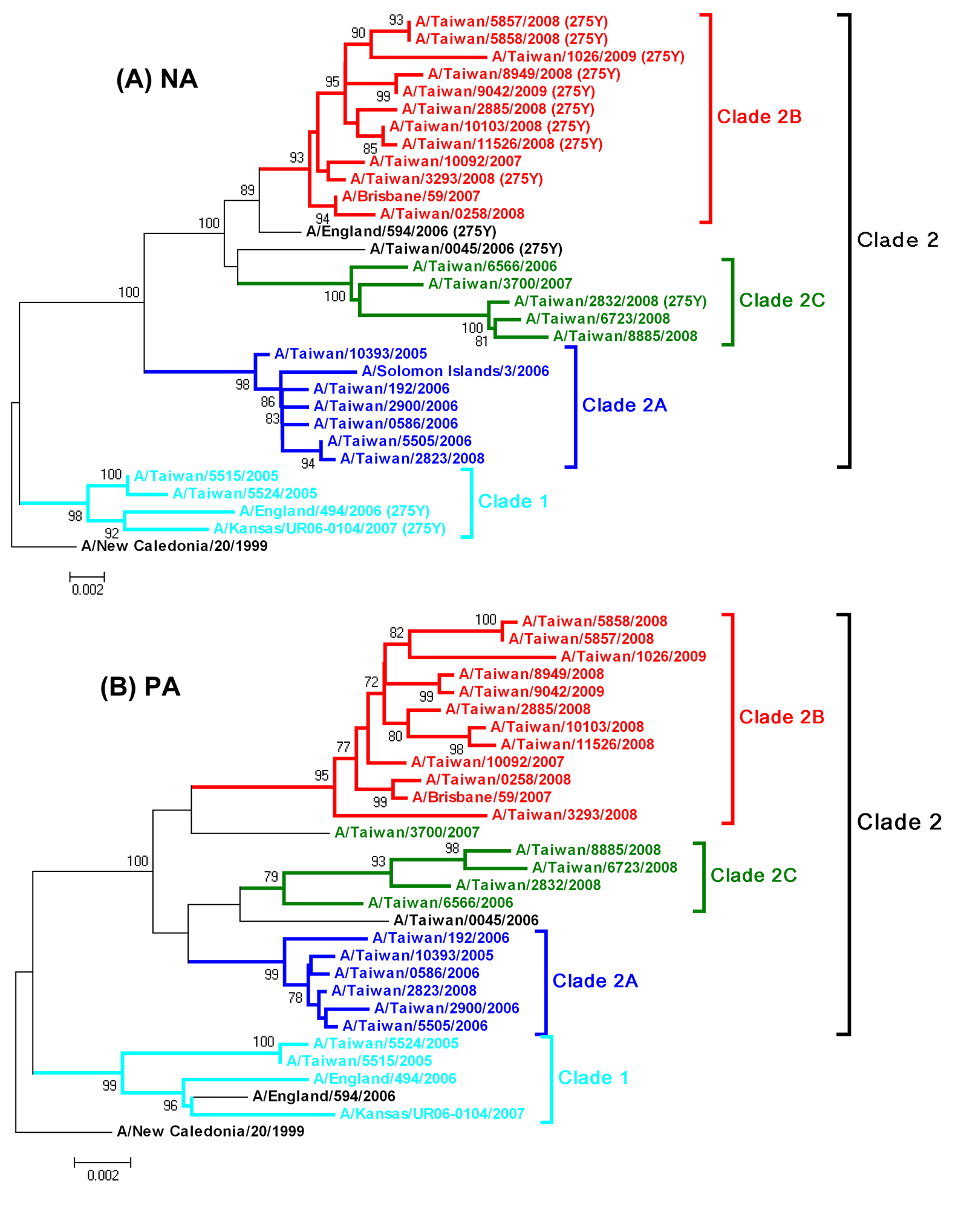

Supplement: Figure S1 — Phylogenetic relationships of the NA and PA segments of influenza A/H1N1 viruses in Taiwan. The phylogenetic analyses were constructed using the neighbor-joining method with 1000 bootstrap replications. Branch values of more than 75 are indicated. All of the phylogenies were rooted with the A/New Caledonia/20/1999. Different clades were shown by different colors. (TIF) [file pone.0018177.s001.tif]

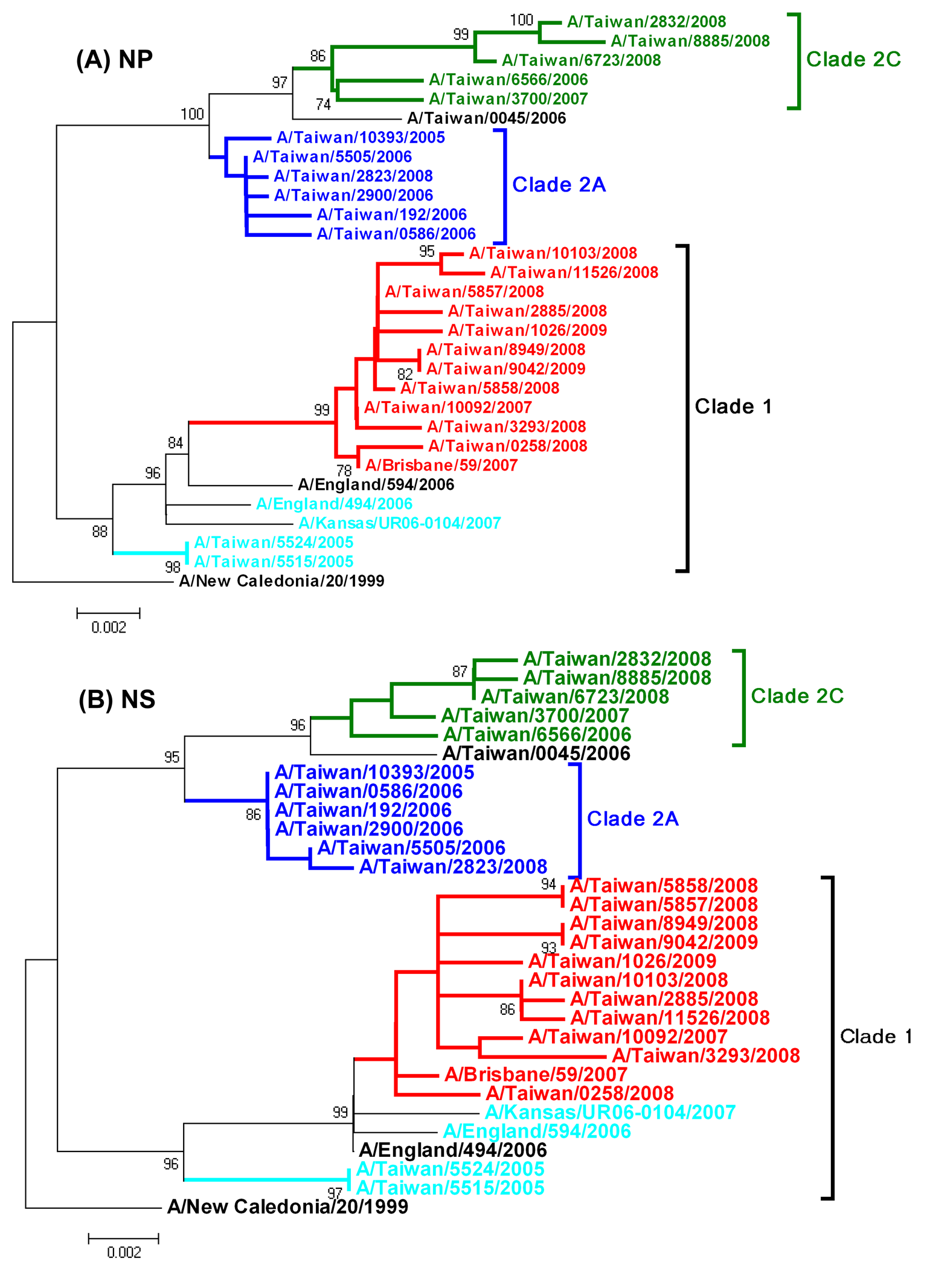

Supplement: Figure S2 — Phylogenetic relationships of the NP and NS segments of influenza A/H1N1 viruses in Taiwan. The phylogenetic analyses were constructed using the neighbor-joining method with 1000 bootstrap replications. Branch values of more than 75 are indicated. All of the phylogenies were rooted with the A/New Caledonia/20/1999. Different clades were shown by different colors. (TIF) [file pone.0018177.s002.tif]
