# Supplementary material for: Reassortment and Mutations Associated with Emergence and Spread of Oseltamivir-Resistant Seasonal Influenza A/H1N1 Viruses in 2005–2009
Source: PLoS One. 2011 Mar 31;6(3):e18177. doi: 10.1371/journal.pone.0018177 (PMC3069057; doi:10.1371/journal.pone.0018177)
Supplement: Table S1 — Amino acid substitutions in various proteins, differing between clades 2B-1 and 2B-2 in the 92 global isolates. (DOC) [file pone.0018177.s003.doc]

Table S1. Amino acid substitutions in various proteins, differing between clades 2B-1 and 2B-2 in the 92 global isolates

| Virus name | Isolation date (y/m/d) | HA | | | | NA | | PB1 | PB1-F2 | | PB2 | |
| --- | --- | --- | --- | --- | --- | --- | --- | --- | --- | --- | --- | --- |
|  |  | 158 | 200 | 202 | 206 | 275 | 354 | 642 | 30 | 41 | 411 | 453 |
| A/Tennessee/UR06-0045/2007 | 2007/1/18 | S | N | G | A | H | D | N | L | H | V | P |
| A/Texas/UR06-0217/2007 | 2007/2/8 | S | N | G | A | H | D | N | L | H | V | P |
| A/Mississippi/UR06-0378/2007 | 2007/2/20 | S | N | G | A | H | D | N | L | H | V | P |
| A/California/UR06-0393/2007 | 2007/2/21 | S | N | G | A | H | D | N | L | H | V | P |
| A/Kentucky/UR06-0476/2007 | 2007/3/1 | S | N | G | A | H | D | N | L | H | V | P |
| A/Brisbane/59/2007 | 2007/7/1 | S | N | G | A | H | D | N | L | H | V | P |
| A/England/545/2007 | 2007/7/28 | S | N | G | A | H | D | N | Q | P | V | P |
| A/Taiwan/71720/2007 | 2007/8/29 | S | N | G | A | H | D | N | R | H | V | P |
| A/England/557/2007 | 2007/11/15 | S | N | G | T | Y | G | S | R | P | I | S |
| A/New Jersey/15/2007 | 2007/11/24 | S | N | G | T | Y | G | S | R | P | I | S |
| A/Hokkaido/07H007/2007 | 2007/12/12 | S | N | G | A | H | D | N | L | H | V | P |
| A/Hyogo/07K030/2007 | 2007/12/18 | S | N | G | A | Y | D | N | L | H | V | P |
| A/England/654/2007 | 2007/12/31 | S | N | G | A | Y | D | N | Q | P | V | P |
| A/England/26/2008 | 2008/1/9 | S | N | G | A | Y | G | N | R | P | I | S |
| A/Washington/AF06/2007 | 2008/1/10 | S | N | G | A | H | D | N | Q | P | V | P |
| A/Nagasaki/07N020/2008 | 2008/1/14 | S | N | G | A | Y | D | N | L | H | V | P |
| A/Taiwan/70132/2008 | 2008/1/16 | S | N | G | A | H | D | N | L | H | V | P |
| A/Taiwan/70167/2008 | 2008/1/21 | S | N | G | A | H | D | N | L | H | V | P |
| A/Japan/AF07/2008 | 2008/1/25 | S | N | G | A | H | D | N | R | P | V | P |
| A/Gunma/07G006/2008 | 2008/1/28 | S | N | G | A | H | D | N | L | H | V | P |
| A/Kyoto/07K316/2008 | 2008/1/28 | S | N | G | A | H | D | N | L | H | V | P |
| A/District of Columbia/WRAMC-1154047/2008 | 2008/2/1 | S | N | G | A | H | D | N | L | H | V | P |
| A/District of Columbia/WRAMC-1154048/2008 | 2008/2/1 | S | N | G | A | H | D | N | L | H | I | P |
| A/Boston/26/2008 | 2008/2/3 | S | N | G | A | H | D | N | L | H | V | P |
| A/Boston/27/2008 | 2008/2/4 | S | N | G | A | H | D | N | L | H | V | P |
| A/Boston/34/2008 | 2008/2/9 | S | N | G | A | H | D | N | L | H | V | P |
| A/Boston/35/2008 | 2008/2/12 | S | N | G | A | H | D | N | L | H | V | P |
| A/Florida/UR07-0022/2008 | 2008/2/14 | S | N | G | A | H | D | N | L | H | V | P |
| A/New Jersey/AF09/2008 | 2008/2/14 | S | N | G | A | H | D | N | L | H | V | P |
| A/Managua/2512.01/2008 | 2008/2/15 | S | N | G | A | H | D | N | L | H | V | P |
| A/Niigata/07F102/2008 | 2008/2/18 | S | N | G | A | H | D | N | R | P | V | P |
| A/Boston/49/2008 | 2008/2/20 | S | N | G | A | H | D | N | L | H | V | P |
| A/Kentucky/UR07-0061/2008 | 2008/2/20 | S | N | G | A | H | D | N | L | H | I | P |
| A/Nagasaki/07N011/2008 | 2008/2/20 | S | N | G | A | H | D | N | L | H | V | P |
| A/Nagasaki/07N035/2008 | 2008/2/20 | S | N | G | A | Y | G | N | R | P | I | S |
| A/Boston/52/2008 | 2008/2/23 | S | N | G | T | Y | G | S | R | P | I | S |
| A/Yokohama/78/2008 | 2008/2/28 | S | N | G | A | Y | G | N | R | P | I | S |
| A/Kyoto/07K454/2008 | 2008/3/3 | S | N | G | A | H | D | N | L | H | V | P |
| A/Managua/1038.01/2008 | 2008/6/8 | S | N | G | A | H | D | N | L | H | V | P |
| A/Managua/1225.02/2008 | 2008/6/8 | S | N | G | A | H | D | N | Q | P | V | P |
| A/Managua/3180.01/2008 | 2008/6/9 | S | N | G | A | H | D | N | L | H | V | P |
| A/Managua/4032.01/2008 | 2008/6/9 | S | N | G | A | H | D | N | L | H | I | P |
| A/Managua/4341.01/2008 | 2008/6/9 | S | N | G | A | H | D | N | L | H | V | P |
| A/Managua/2055.01/2008 | 2008/6/10 | S | N | G | A | H | D | N | L | H | V | P |
| A/Managua/156.01/2008 | 2008/6/11 | S | N | G | A | H | D | N | Q | P | V | P |
| A/Managua/4070.01/2008 | 2008/6/11 | S | N | G | A | H | D | N | L | H | I | P |
| A/Managua/4017.02/2008 | 2008/6/20 | S | N | G | A | H | D | N | L | H | V | P |
| A/Managua/3027.01/2008 | 2008/6/24 | S | N | G | A | H | D | N | L | H | V | P |
| A/Managua/4086.02/2008 | 2008/6/25 | S | N | G | A | H | D | N | L | H | V | P |
| A/Managua/3570.01/2008 | 2008/6/27 | S | N | G | A | H | D | N | Q | P | V | P |
| A/Managua/954.02/2008 | 2008/7/1 | S | N | G | A | Y | D | N | L | H | V | P |
| A/Managua/5214.01/2008 | 2008/7/25 | S | N | G | A | H | D | N | Q | P | V | P |
| A/Managua/5007.01/2008 | 2008/8/7 | S | N | V | T | Y | G | S | R | P | V | S |
| A/Managua/496.01/2008 | 2008/8/28 | S | N | V | T | Y | G | S | R | P | V | S |
| A/Managua/4924.01/2008 | 2008/10/6 | S | N | V | T | Y | G | S | R | P | V | S |
| A/Managua/2860.01/2008 | 2008/10/11 | S | N | G | T | H | D | N | L | H | V | P |
| A/Managua/3418.01/2008 | 2008/10/11 | S | N | G | T | H | D | N | L | H | V | P |
| A/Managua/1985.01/2008 | 2008/10/20 | S | N | V | T | Y | G | S | R | P | V | S |
| A/Managua/4315.04/2008 | 2008/10/25 | S | N | G | T | H | D | N | L | H | V | P |
| A/Managua/309.01/2008 | 2008/10/30 | S | N | G | T | H | D | N | L | H | V | P |
| A/Managua/4412.01/2008 | 2008/11/6 | S | N | G | T | H | D | N | L | H | V | P |
| A/Managua/5751.01/2008 | 2008/11/11 | S | N | G | T | H | D | N | L | H | V | P |
| A/Boston/12/2008 | 2008/12/4 | S | N | G | A | H | D | N | L | H | V | P |
| A/Tottori/08T010/2008 | 2008/12/31 | N | N | A | T | Y | G | S | R | P | I | S |
| A/Shanghai/LWS1/2009 | 2009/1/3 | N | N | A | T | Y | G | S | R | P | I | S |
| A/Gunma/08G006/2009 | 2009/1/5 | S | N | V | T | Y | G | S | R | P | V | S |
| A/Kyoto/08K056/2009 | 2009/1/7 | S | N | G | T | Y | G | N | R | P | I | S |
| A/Nagasaki/08N006/2009 | 2009/1/10 | S | N | V | T | Y | G | S | R | P | V | S |
| A/Hokkaido/08H024/2009 | 2009/1/13 | N | N | A | T | Y | G | S | Q | P | I | S |
| A/Niigata/08F031/2009 | 2009/1/14 | N | N | A | T | Y | G | S | R | P | I | S |
| A/Niigata/08F093/2009 | 2009/1/19 | N | N | A | T | Y | G | S | R | P | I | S |
| A/Niigata/08F188/2009 | 2009/1/26 | N | N | A | T | Y | G | S | R | P | I | S |
| A/California/VRDL134/2009 | 2009/4/27 | S | S | S | T | Y | G | N | R | P | I | S |
| A/California/VRDL135/2009 | 2009/4/27 | N | N | A | T | Y | G | S | R | P | I | S |
| A/California/VRDL140/2009 | 2009/4/27 | S | N | V | T | Y | G | S | R | P | V | S |
| A/New York/1692/2009 | 2009/4/27 | S | N | V | T | Y | G | S | R | P | V | P |
| A/New York/3052/2009 | 2009/4/27 | S | N | V | T | Y | G | S | R | P | V | P |
| A/New York/3150/2009 | 2009/4/27 | S | N | V | T | Y | G | S | R | P | V | S |
| A/California/VRDL141/2009 | 2009/4/28 | S | S | S | T | Y | G | N | R | P | I | S |
| A/New York/3095/2009 | 2009/4/29 | S | S | S | T | Y | G | N | R | P | I | S |
| A/New York/3315/2009 | 2009/4/29 | S | S | S | T | Y | G | N | R | P | I | S |
| A/New York/3442/2009 | 2009/5/9 | N | N | A | T | Y | G | S | R | P | I | S |
| A/New York/3467/2009 | 2009/5/13 | S | N | V | T | Y | G | S | R | P | V | P |
| A/New York/3768/2009 | 2009/5/26 | S | N | V | T | Y | G | S | R | P | V | P |
| A/Thailand/CU-B42/2009 | 2009/6/16 | N | N | A | T | Y | G | S | R | P | I | S |
| A/Thailand/CU-H17/2009 | 2009/6/18 | N | N | A | T | Y | G | S | L | H | I | S |
| A/Thailand/CU-B97/2009 | 2009/6/22 | N | N | A | T | Y | G | S | R | P | I | S |
| A/Thailand/CU-B267/2009 | 2009/7/2 | N | N | G | T | Y | G | S | R | P | I | S |
| A/Thailand/CU-B589/2009 | 2009/7/10 | N | N | A | T | Y | G | S | R | P | I | S |
| A/Thailand/CU-B685/2009 | 2009/7/12 | N | N | A | T | Y | G | S | R | P | I | S |
| A/Thailand/CU-H223/2009 | 2009/7/16 | N | N | A | T | Y | G | S | R | H | I | S |
| A/Thailand/CU-H565/2009 | 2009/9/2 | N | N | A | T | Y | G | S | R | H | I | S |
